# Outcome of Immediate Dose Optimization of Infliximab in Inflammatory Bowel Disease Patients

**DOI:** 10.1093/ecco-jcc/jjab207

**Published:** 2021-11-18

**Authors:** Péter Bacsur, Klaudia Farkas, Tamás Molnar

**Affiliations:** Department of Medicine, Szent-Györgyi Albert Medical School, University of Szeged, Szeged, Hungary; Department of Medicine, Szent-Györgyi Albert Medical School, University of Szeged, Szeged, Hungary; Department of Medicine, Szent-Györgyi Albert Medical School, University of Szeged, Szeged, Hungary

Dear Editors,

We read with interest the article by Bossuyt *et al*. comparing the impact of an ultra-proactive therapeutic drug monitoring [TDM] algorithm based on point-of care testing [POCT] with a reactive strategy on long-term outcome of inflammatory bowel disease [IBD] patients.^1^ Reactive measurement of infliximab [IFX] trough levels is associated with better outcome, while proactive measurement has not yet lived up to expectations. Ultra-proactive TDM based on POCT and ad-hoc dose adjustment could provide personalized dosing of IFX.^2^ A treat-to-target [T2T] approach has been accepted in the management of IBD.^3^ However, conflicting evidence and recommendations are available according to the merit of using TDM in the IBD setting, although declining serum drug concentrations before the development of symptoms provide a good candidate for treatment adjustment to avoid relapse. Bossuyt *et al*. concluded that treatment optimization with IFX should be based on strict clinical, biochemical and endoscopic follow-up, with TDM as an adjuvant in a selected population. Similarly, we were interested in whether POCT-based measurement of IFX in combination with the POCT-based assessment of C-reactive protein [CRP] and faecal calprotectin [FC] would change our clinical practice and which of these parameters would best guide decision-making.

**Figure 1.**
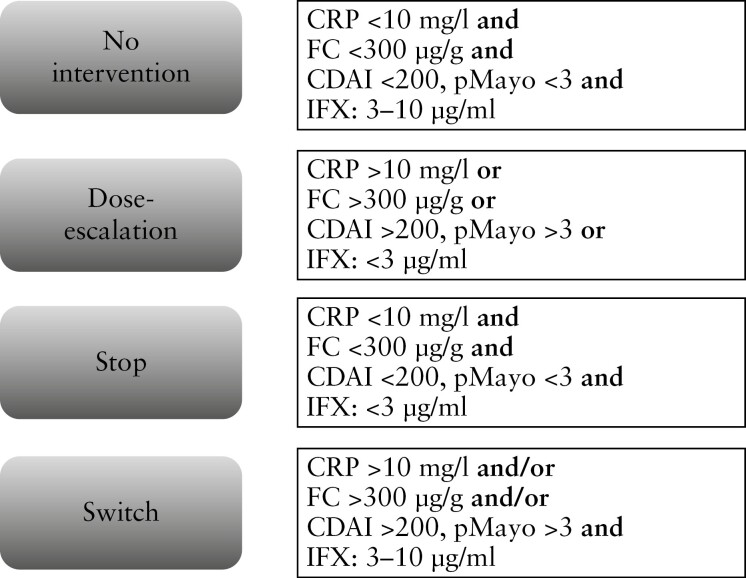
Patient groups according to clinical and biochemical parameters.

Twenty-six Crohn’s disease [CD] and 21 ulcerative colitis [UC] patients were enrolled in the study. Blood and faecal samples were obtained from the patients on the day of the subsequent IFX infusion. CRP, FC and serum IFX were measured via POCT lateral flow assays. Crohn’s disease activity index [CDAI] and pMayo scores were calculated. Patients were assigned to four groups according to these results [Figure 1].

Based on the POCT, dose intensification [DI] was performed in 27 patients, no intervention [NI] in 13 patients and therapy stopped [ST] in six patients. The mean serum level of IFX increased [*p* < 0.01], while CDAI [*p* = 0.027], pMayo [*p* = 0.012] decreased at month 6 and CRP [*p* = 0.021] decreased to month 4 compared to baseline. One patient was switched to ustekinumab and adalimumab at month 2. Except for one patient in the NI group, all remained in remission at month 6. One patient in the ST group required reinduction of therapy with adalimumab at month 2; the other five patients were in sustained remission. In summary, a change in therapy was implemented in 34 cases based on benchmarked concentrations of serum IFX [20×] and FC [5×]. Symptoms improved in 90% of patients, and serum IFX concentration increased in 87% of patients after dose intensification. Our personal experience revealed that therapeutic interventions were performed more easily and earlier when being aware of drug levels.

Our results highlighted the importance of TDM in a T2T approach and suggested at least short-term benefits of using rapid tests in daily practice; nevertheless there are additional costs of this proactive monitoring strategy. POCT measurements could help physicians to make established decisions in the spirit of T2T with the advantage of avoiding delayed decision-making and better adherence to the therapy by patients.
